# Diagnostic markers based on a computational model of lipoprotein metabolism

**DOI:** 10.1186/2043-9113-1-29

**Published:** 2011-10-26

**Authors:** Daniël B van Schalkwijk, Ben van Ommen, Andreas P Freidig, Jan van der Greef, Albert A de Graaf

**Affiliations:** 1 TNO Quality of Life, Business Unit Biosciences, Zeist and Leiden, the Netherlands; 2 Leiden Amsterdam Centre for Drug Research (LACDR), Analytical Sciences division, Leiden, the Netherlands; 3 The Netherlands Bioinformatics Centre (NBIC), Nijmegen, the Netherlands; 4 Amsterdam Molecular Therapeutics (AMT), Amsterdam, the Netherlands

## Abstract

**Background:**

Dyslipidemia is an important risk factor for cardiovascular disease and type II diabetes. Lipoprotein diagnostics, such as LDL cholesterol and HDL cholesterol, help to diagnose these diseases. Lipoprotein profile measurements could improve lipoprotein diagnostics, but interpretational complexity has limited their clinical application to date. We have previously developed a computational model called Particle Profiler to interpret lipoprotein profiles. In the current study we further developed and calibrated Particle Profiler using subjects with specific genetic conditions. We subsequently performed technical validation and worked at an initial indication of clinical usefulness starting from available data on lipoprotein concentrations and metabolic fluxes. Since the model outcomes cannot be measured directly, the only available technical validation was corroboration. For an initial indication of clinical usefulness, pooled lipoprotein metabolic flux data was available from subjects with various types of dyslipidemia. Therefore we investigated how well lipoprotein metabolic ratios derived from Particle Profiler distinguished reported dyslipidemic from normolipidemic subjects.

**Results:**

We found that the model could fit a range of normolipidemic and dyslipidemic subjects from fifteen out of sixteen studies equally well, with an average 8.8% ± 5.0% fit error; only one study showed a larger fit error. As initial indication of clinical usefulness, we showed that one diagnostic marker based on VLDL metabolic ratios better distinguished dyslipidemic from normolipidemic subjects than triglycerides, HDL cholesterol, or LDL cholesterol. The VLDL metabolic ratios outperformed each of the classical diagnostics separately; they also added power of distinction when included in a multivariate logistic regression model on top of the classical diagnostics.

**Conclusions:**

In this study we further developed, calibrated, and corroborated the Particle Profiler computational model using pooled lipoprotein metabolic flux data. From pooled lipoprotein metabolic flux data on dyslipidemic patients, we derived VLDL metabolic ratios that better distinguished normolipidemic from dyslipidemic subjects than standard diagnostics, including HDL cholesterol, triglycerides and LDL cholesterol. Since dyslipidemias are closely linked to cardiovascular disease and diabetes type II development, lipoprotein metabolic ratios are candidate risk markers for these diseases. These ratios can in principle be obtained by applying Particle Profiler to a single lipoprotein profile measurement, which makes clinical application feasible.

## Background

Dyslipidemia is an important risk factor for cardiovascular disease and type II diabetes. Especially low-density lipoprotein (LDL) cholesterol and LDL particle concentrations are known to be positively associated with cardiovascular disease risk [1], and reaching low LDL cholesterol concentrations is a primary goal for therapy [2]. Other recognized markers for metabolic syndrome include triglycerides and HDL cholesterol [2]. LDL particles contain the protein apoB, and are to a large extent a metabolic product of the larger apoB-containing lipoproteins, very low density lipoproteins (VLDL), and intermediate-density lipoproteins (IDL). Technological advances allow the full size spectrum of lipoproteins to be measured in increasing detail [3-7], creating a detailed lipoprotein profile. Although such a profile contains much information, it has not led to a single diagnostic value that is easily applicable. The detailed lipoprotein profile needs a further interpretation and validation to be useful for the clinic. One example of interpreting this detailed data is the pooling of all LDL particles, and reporting an 'LDL particle number'. This diagnostic has proven to be successful at predicting cardiovascular risk [1]. Still, the detailed lipoprotein profiles contain more information that is discarded when only reporting LDL particles. A computational model that can characterize the state of metabolic processes affecting lipoproteins, based on the additional information contained in a lipoprotein profile, may be of added value in the clinic.

Lipoprotein metabolism of VLDL, IDL, and LDL comprises three main processes. The lipoproteins are produced by the liver, then lose triglycerides through lipolysis and are finally taken up from the bloodstream by the liver. The lipolysis process occurs in extrahepatic tissues through lipoprotein lipase (LPL), which mainly affects the larger very-low-density lipoproteins (VLDL) [8], whereas in the liver lipolysis occurs through hepatic lipase (HL), mainly affecting the smaller IDL and LDL [9,10]. LPL activity is also known to affect HDL metabolism [11]. Measuring the rates of these processes is generally carried out using stable-isotope or radioactive-isotope tracer techniques. The most popular approaches perform kinetic tracer analysis of the large constituent protein apolipoprotein B to obtain lipoprotein fluxes [12]. These techniques are costly and labor-intensive. A good characterization of the status of lipoprotein metabolism is, therefore, an extensive and difficult procedure at this time.

**Figure 1:**
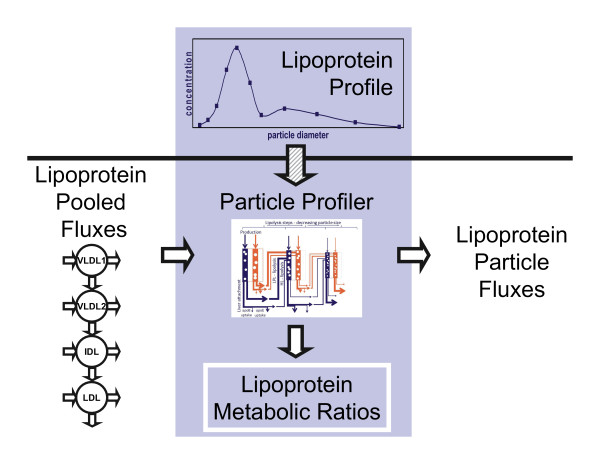
**Data use and generation in current and future model implementations**. In the current study, we used Particle Profiler as indicated below the vertical bar. Pooled lipoprotein flux data was used for fitting the model to data of individual subjects, and the fitted model was used to generate lipoprotein particle flux data and lipoprotein metabolic ratios. The light blue area illustrates the final application we aim at. In that application, Particle Profiler will be applied to lipoprotein profile data, which allows for the quantification of lipoprotein metabolic ratios only.

Since it would be helpful to get an impression of lipoprotein metabolism in a fast and less laborious way, we have developed a computational model called Particle Profiler [13,14]. This model was designed to derive ratios between the various lipoprotein metabolic processes, such as the ratio between lipolysis and production, from a single lipoprotein profile. Figure 1 shows how in the model development phase, reported in the current study, we chose to derive particle-based lipoprotein fluxes and lipoprotein metabolic ratios from previously published pooled lipoprotein flux data (see *e.g*. [15]). This 'pooled lipoprotein flux data' includes particle concentrations and fluxes (production, lipolysis and uptake) in four size classes: VLDL1, VLDL2, IDL and LDL. Figure 1 also shows that the future application to lipoprotein profiles will not be able to produce particle-based lipoprotein fluxes, but only lipoprotein metabolic ratios. It is impossible to obtain the absolute fluxes, since the lipoprotein profile measurements are taken from a single blood sample and do not contain kinetic information. Still, the metabolic ratios show whether metabolic processes are well balanced or not, which could give an indication of health status.

Clinical application of the previously published Particle Profiler model [13] requires further model development and calibration, as well as both technical and clinical validation. Model development and calibration are necessary to overcome previously identified shortcomings (see model development below). Technical validation needs to ensure that the model is able to accurately reflect lipoprotein metabolism, as measured by experiment in a wide range of subjects. In the model, the metabolic rate of a particle depends on its size. Using the metabolic rate information of each particle, the model can calculate the average metabolic rate of particles in a certain size range of interest, for instance the VLDL size range (see [13]). The model also distinguishes different metabolic routes, such as particle lipolysis through LPL or HL. It is impossible to measure these quantities directly. Instead, a feasible approach to technical validation is to calibrate the model with pooled lipoprotein flux data from genetically deficient subjects, and subsequently corroborate it with pooled lipoprotein flux data from a range of different normolipidemic and dyslipidemic subjects. Calibration and subsequent corroboration with pooled lipoprotein flux data is the only available route of technical validation. Subsequent steps of clinical validation should point out whether the values produced by Particle Profiler correctly inform about disease status.

In this study we address two questions. First, whether a further developed and calibrated Particle Profiler model could be corroborated with pooled lipoprotein flux data from a range of different normolipidemic and dyslipidemic subjects. Second, whether Particle Profiler- based ratios of VLDL metabolic processes derived from pooled lipoprotein flux data indicate relevant differences between dyslipidemic and normolipidemic subjects. Continuing on from the second question, we also examined the effect of statin and fibrate treatment on the VLDL metabolic ratios.

## Results

### Algorithm development

The initial Particle Profiler model [13]^1 ^includes functions that specify the following processes: production, liver attachment, lipolysis through a hepatic HL-related process and an extrahepatic LPL-related process, and uptake through an apoB and an apoE-related process. Liver attachment is immediately followed either by HL-related lipolysis or one of the uptake processes. The model includes VLDL, IDL and LDL particles.

The mathematical functions describing liver uptake and lipolysis needed further development for two reasons. First of all, for three out of sixteen analyzed subjects in our first paper, the model was not able to reproduce the lipoprotein fluxes well. The deviation was mainly due to the uptake fluxes, suggesting that the mathematical functions used to model uptake processes were suboptimal. Secondly, a parameter identifiability analysis, using the covariance matrix produced by the parameter fitting routine (data not shown), showed that detailed lipoprotein profiles, in contrast to lipoprotein kinetics data, do not contain enough information to fit the six parameters in the original model. Because of problem for future model applications to lipoprotein profile data, we decided to reduce the dimensionality of the model by one parameter to five parameters through simplifying the hepatic lipolysis function. We expect that this necessary simplification wil reduce model performance, but by smart reduction and subsequent calibration we attempt to limit the performance reduction. Since the both the uptake and hepatic lipase functions relate to liver processes, we introduced new functions for lipoprotein attachment to the liver, and lipoprotein lipolysis and uptake by the liver.

The new model of liver-related aspects of lipoprotein metabolism describes the biological process as two phases, similar to the earlier model. In the first phase, the particle is attached to the liver via either apoB- or apoE-related mechanisms. In the second phase, particles attached through the apoB-related mechanism are directly taken up, whereas particles attached through the apoE-related mechanism can be either taken up or lipolyzed. The probability that a particle is taken up or lipolyzed depends on the size of the particle, with larger particles having a greater probability of being taken up instead of lipolyzed [13].

The full development of the new functions is described in Additional file 1; all symbols used in the equations in this paper are defined in Table 1. The new function describing how the liver attachment rate *k_a, liver _*varies with particle size *d *is based on the Weibull distribution; the Rayleigh distribution was used in the previous implementation [13]. The main advantage of the Weibull function is its ability to take on different shapes, which can be fine-tuned better to match the observed liver uptake. The new function is given by *(eq. 1)*:

**Table 1:** Overview of state variables, variables, parameters and constants used in this paper

		State Variables - determine the system state
*d_i, j_*	nm	Lipoprotein particle diameter in the *i-*th step of a lipolysis cascade, in the *j*-th subclass of the size range covered by that cascade step
*Q*_*ss *_(*d_i, j_*)	Particles * dL^-1^	Steady-state particle pool size in a pool with mean particle diameter *d_i, j _*
		Variables - specify processes and output
$d_{i,j}^{r}$	nm	the radius of the subclass with average diameter *d_i, j_*
*J_l _*(*d_i, j_*)	Particles * dL^-1 ^* day^-1^	Particle flux into the pool with mean particle diameter *d_i, j _*due to extrahepatic lipolysis
*J_l, liver _*(*d_i, j_*)	Particles * dL^-1 ^* day^-1^	Particle flux into the pool with mean particle diameter *d_i, j _*due to hepatic lipolysis
*k_l_*(*d*)	day^-1^	Particle size dependent extrahepatic lipolysis rate
*k_a, liver _*	day^-1^	Particle size dependent liver attachment rate (attachment is followed by either lipolysis or uptake)
*k_l, liver _*(*d*)	day^-1^	Particle size dependent liver lipolysis rate
*k_u, liver _*(*d*)	day^-1^	Particle size dependent liver uptake rate
*n_tg _*(*d_i, j_*)	Molecules * particle^-1^	Number of triglyceride molecules in a lipoprotein particle with diameter *d_i, j_*
Q_*_	Particles * dL^-1^	Steady-state particle pool size in the size range called *
$\bar{k_{u,liver}^{*}}$	day^-1^	Particle size dependent liver uptake rate, averaged per particle over the size range called *
$\bar{k_{l}^{}}$	day^-1^	Particle size dependent extrahepatic lipolysis rate, averaged per particle over all particles in the model
$\bar{k_{a,liver}^{}}$	day^-1^	Particle size dependent liver attachment rate, averaged per particle over all particles in the model
J_p,* _	Particles * dL^-1^*day^-1^	Particle production flux into the size range called *
J_in, *_	Particles * dL^-1 ^* day^-1^	Particle production influx (production + lipolysis) into the size range called *
*Q*_*out*_ ([*d_a _d_b_*])	Particles * dL^-1^	Steady state particle pool size in interval from *d_a _*to*d*_b_in the final particle concentration profile.
		Parameters - are optimized using data
*k_l, max_*	day^-1^	maximum rate at which extrahepatic lipolysis takes place
*k_a, apoEmax_*	day^-1^	maximum rate at which liver binding mediated by ApoE takes place
*k_a, apoB_*	day^-1^	rate at which liver binding mediated by ApoB takes place
*A*	nm	shape parameter for liver binding mediated by ApoE
*B*	-	shape parameter for liver binding mediated by ApoE
*σ_u, liver _*	nm	shape parameter describing fraction of liver attachment which is taken up (instead of lipolyzed) - with changing particle size
		Model constants and derived variables - calibrated in this paper
*d_a, apoEmin_*	17 nm	minimum particle diameter at which liver binding mediated by ApoE takes place
d_hl, peak_	31.33 nm	Hepatic lipase lipolysis peak size (see Eq. 5)
*s_u, liver _*	7.87	Liver uptake shape constant (see Eq. 2)
*d_l, min_*	25.13 nm	minimum size at which extrahepatic lipolysis occurs (see Eq. 4 in [13])
*σ_l_*	77.35 nm	shape constant for extrahepatic lipolysis (see Eq. 4 in [13])

*for d *<*d*_*a, apoE *min_

ka,liver(d)=ka,apoE max⋅⋅(d-da,apoE min)B-1e-d-da,apoE minABeB-1B⋅lnB-1B-1⋅AB-1++ka,apoB

*for d *<*d*_*a, apoE *min_

$$k_{u,liver}\left(d\right)=k_{a,apoB}$$

Where *k*_*a, liver *max _is the maximum liver attachment rate, *k_a, apoB _*is the apoB-related liver attachment rate, *d*_*a, apoE *min _is the minimum particle size at which apoE-related liver attachment takes place, and A and *B *are shape parameters.

The model specifies that once a particle has been attached it is either directly taken up or lipolyzed. In general larger particles are lipolyzed more often, and smaller particles are taken up more often, although the exact rates differ per individual. The function describing how the lipolysis/uptake ratio varies with particle size also was a Rayleigh distribution in the previous model implementation. In the new model implementation this function is described using a Weibull distribution. The new equation for liver uptake, modeled as liver attachment followed by uptake, is given by *(eq. 2)*:

For *d *<*d*_*a, apoE *min_

ku,liver(d)=ka,liver(d)-ka,apoB⋅⋅1-e-d-da,apoE minsu,liver⋅σu,liversu,liver++ka,apoB

For *d *<*d*_*a, apoE *min_

$$k_{u,liver}\left(d\right)=k_{a,apoB}$$

Where all symbols have the same meaning as before, and *s_u, liver _*is a liver uptake constant, that helps to determine the shape of the uptake function. The Weibull function normally has two shape parameters, but the available data do not contain enough information to fit both. Therefore we gave *s_u, liver _*a constant value that does not vary between patients, but that we optimize under 'model calibration'. *σ_u, liver _*is a liver uptake shape parameter that does vary between patients and can be adjusted in parameter optimization.

Since in the model, the attached particles that are not taken up are lipolyzed, the equation for liver lipolysis *k_l, liver _*is:

(3) $$k_{l,liver}\left(d\right)=k_{a,liver}\left(d\right)-k_{u,liver}\left(d\right)$$

The figures in Additional file 1 show the new version of the liver attachment, lipolysis and uptake functions. In the methods section we define the *d_hl, peak _*constant that describes the particle size at which hepatic lipase activity is at its peak. By fixing this constant, we reduced the free parameters in the model from six to five. Table 1 gives an overview of what parameters were optimized (fitted) using the patient's data in the current implementation.

### Model Calibration

The model equations contain parameters that are allowed to assume different values for different patients and model constants that are fixed to the same value for all patients. The model constants contain the biological information that, for instance, allows the model to distinguish hepatic lipolysis from extrahepatic lipolysis. Therefore, it is very important that these constants have the correct values. The constants optimized here are: *d_hl, peak_*, *s_u, liver_*, *σ_a, lpl _*and *d*_*a, lpl *min_, which are related to HL lipolysis, liver uptake and LPL lipolysis (2 constants) respectively (see Table 1 for an overview of all notation). The first two constants are new to the model, the last two were already present in the first version [13], but are now given new values. To estimate the model constants, one needs data from subjects in which particular process stands out clearly. Below, we first describe what data we used to estimate specific constants, and in continuation we describe how the constants were estimated.

To estimate the HL-related model constant *d_hl, peak_*, which indicates the lipoprotein particle size at which HL activity is highest, we used patients with lipoprotein lipase (LPL) deficiency. In these patients the only remaining lipolysis activity is due to HL. Data on lipoprotein metabolic fluxes in such patients came from [16].

To estimate the model constant *s_u, liver_*, which helps to model the liver uptake rate at different lipoprotein particle sizes, subjects are needed in which uptake processes take place with least interference from lipolysis processes. By inspecting the kinetic data, we found that normolipidemic ApoE 3/3 subjects meet these criteria best. Therefore, *s_u, liver _*was estimated using data on lipoprotein metabolic fluxes in ApoE 3/3 subjects from [17].

To estimate model constants related to LPL lipolysis, subjects with the ApoE 2/2 genotype were used. Subjects with the ApoE 2/2 isoform are known to have impaired uptake of large VLDL and chylomicron particles [17]. Since VLDL particles can either be taken up by the liver or lipolyzed by LPL, an impaired uptake means that the LPL lipolysis process, that mainly takes place in the VLDL size range, can be distinguished clearly. Also, the lipolysis of smaller particles was found to be impaired in ApoE 2/2 subjects [17], indicating a less effective hepatic lipase function, which should make the LPL activity even more clearly discernable, also for smaller particles. Therefore, the data from subjects with the ApoE 2/2 phenotype were useful for estimating two model constants related to LPL: *σ_a, lpl _*and *d*_*a, lpl *min_. Because in apoE 2/2 patients hepatic lipase function is inhibited, the model needed to be adjusted slightly. We allowed the 'HL peak size' (*d_hl, peak_*) parameter to be optimized for each individual apoE 2/2 subject, which is otherwise constant for all subjects. In this way the model could better handle the special condition of very low HL activity.

In order to determine the model constants via parameter fitting, a double-layered fitting routine was constructed. On the first layer, the algorithm searched for the optimal value for the model constant. The second layer of the routine fitted the parameters of the model at each selected constant value. Both layers used the Levenberg-Marquardt algorithm as implemented in MATLAB's nlinfit method of version 7.7.0 (R2008b) for fitting the constants and parameters respectively. The used error functions can be found in the methods section. Parameter identifiability was inspected using the covariance matrix produced by the fitting routine. In this way, the model parameters were estimated per individual, while the model constants were estimated for the whole population, using a group of patients judged most suited for the determination of that constant.

We chose to fit the constants in the same order as they were discussed above: first hepatic lipase constants, then liver uptake constants, and finally LPL lipolysis constants. Each time the newly found constant value was used in the process of fitting the subsequent constants. The order of fitting and the type of subjects, discussed above, were chosen in such a way that the constants that had not yet been fitted exerted minimal influence on the constant being fitted. For instance, the LPL deficient patients used for determining the Hepatic Lipase constants have little LPL activity and little liver uptake activity related to the unknown uptake constants.

The model constants obtained from the optimization process are given in Table 1. The constants show that Hepatic Lipase activity is highest in particles around 31 nm in size, which is the IDL and small VLDL2 size range. Instead, LPL lipolysis affects particles of approximately 25 nm (lower cutoff) and higher, but the large shape parameter *σ_l _*indicates that the LPL rate very gradually increases with size, and really becomes important for the larger VLDL2 and the VLDL1 particles, which LPL is known to affect more. The translation into exact metabolic rates depends on the model parameters that differ for every subject (shown in Table 1).

## Comparison with earlier results

With the new model equation and settings, we examined whether the results are comparible to those in our first paper [13]. The subjects reported by Packard et al. [18] were fitted with the new model and the results were compared with those from the first model implementation [13]. In the first implementation, the model reproduced a shift in 'LDL peak size', which was independently measured. The model analysis also identified credible changes in relevant biological processes between groups with differing 'LDL peak size'. For the new model, we examined how well the data was fitted, whether the shift in 'LDL peak size' was still reproduced by the model, and whether the model still identified similar differences in biological processes between groups with differing 'LDL peak size'. Since the models are different and the new model has one free parameter less, which in general leads to less optimal model performance, we did not expect an exact match between the model results. This comparison does show what similarities and differences exist.

The model with optimized constants and one free parameter less than in the first implementation [13] reproduced the data from Packard *et al*. [18] well. The overall fit error, defined in [13], was 7.3% ± 3.6% in this study compared to a 7.2% ± 4.5% error in our first paper. In [13] the model calculated a difference in LDL size of 4.2 nm between subjects with phenotype 'A' (large LDL particles) versus phenotype ' B' (small LDL particles). This LDL size difference was significant, with a Kruskal-Wallis p-value of 0.026. In the new implementation we saw a similar difference, although with 1.9 nm it was less pronounced. The Kruskal-Wallis test indicated a trend, with p = 0.089. The most likely cause of this difference is the description of hepatic lipolysis, which lost one free parameter in the new model. This conjecture was confirmed by studying the difference in metabolic processes between subjects with phenotypes 'A' and 'B' from [18], as analyzed by our initial model versus the new implementation. The latter are shown in Table 2. As observed earlier [13], we saw differences in the average particle lipolysis rate in VLDL 1 and VLDL 2, and in the average particle uptake rate in LDL between 'A' and 'B' phenotypes. In contrast to our first study [13], no differences between the two phenotypes were found for HL lipolysis in LDL. Instead differences were found in uptake of IDL particles and LPL lipolysis of VLDL and IDL. For particle age similar differences between 'A' and 'B' phenotypes were found in LDL, VLDL2 and VLDL1, as well as an additional difference in IDL age. For particle size differences between phenotypes were found for LDL and IDL, and for the new implementation an additional difference in VLDL1 particle diameter was found. Overall this comparison indicates that the new implementation made the model less sensitive to changes in LDL metabolism, but increased its power to identify changes in LPL lipolysis in the VLDL and IDL range, while the overall model fitting performance did not change. So all in all we have carried out a necessary simplification of the model, while keeping the overall model performance stable.

**Table 2:** Comparison with results of first paper [13]

		Significance of inter-group difference	Significance of inter-group difference
	units	p-value [13]	p-value current
Size-specific process indicator parameters			
Average particle lipolysis rate LDL	day^-1^	0.026	N.S.
Average particle lipolysis rate VLDL2	day^-1^	0.026	0.014
Average particle lipolysis rate VLDL1	day^-1^	0.005	0.007
Average particle LPL lipolysis rate IDL	day^-1^	N.S.	0.005
Average particle LPL lipolysis rate VLDL2	day^-1^	N.S.	0.006
Average particle LPL lipolysis rate VLDL1	day^-1^	N.S.	0.006
Average particle uptake rate LDL	day^-1^	0.042	0.052
Average particle uptake rate IDL	day^-1^	N.S.	0.042
Average particle HL attachment rate LDL	day^-1^	0.026	N.S.
Average particle HL attachment rate VLDL2	day^-1^	0.034	N.S.
Size and age parameters			
Average particle age LDL	hours	0.014	0.031
Average particle age IDL	hours	N.S.	0.033
Average particle age VLDL2	hours	0.026	0.025
Average particle age VLDL1	hours	0.026	0.022
Average particle diameter LDL	nm	0.027	0.089
Average particle diameter IDL	nm	0.039	0.026
Average particle diameter VLDL1	nm	N.S.	0.045

Significance of difference between groups with lipoprotein phenotypes A (LDL peak size < 25 nm), I (25 nm < LDL peak size < 26 nm) and B (LDL peak size > 26 nm) from [18] for size-specific indicator parameters. The results from the further developed and calibrated model versus the original model from are shown [13]. Only those processes that show a significant difference (p < 0.1) using the nonparametric Kruskal-Wallis test are included.

### Testing

#### Model corroboration

Particle Profiler calculates metabolic process rates averaged per particle, and distinguishes between HL and LPL lipolysis. Because these quantities cannot be measured directly, the best way of validating the model is through corroboration. Patients with different metabolic conditions need to be modeled equally well. Therefore, we applied Particle Profiler to lipoprotein kinetics data from several studies [16-26] containing subjects with a wide range of dyslipidemias that the model had not yet analyzed before. We inspected whether the model was able to fit data from all these studies well. If successful, this indicates that the biology incorporated in the model in the form of mathematical equations is adequate to describe the measured experimental data, or in other words that the model is consistent with reality.

The overall fit error for the data from the different lipoprotein kinetics studies was 10.4% with a standard deviation of 6.5%. The highest errors were found when analyzing data from Demant et al. 1998 [21], where the fit error was 20.1% ± 6.4%. Many subjects in this study, including the controls, had a high VLDL2 pool that our model was unable to reproduce in conjunction with the reported flux values. We consider the deviation in this single study of small relevance, because it occurs not only in the diseased subjects, but also in the controls. Normolipidemic controls do not give problems in any other study. Without these subjects the average fit error was 8.8% ± 5.0%.

### Implementation

**Figure 2:**
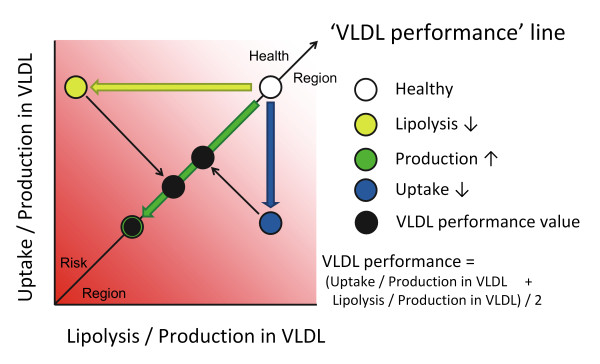
**Graphical representation of the VLDL performance diagnostic**. When applying the Particle Profiler model to a lipoprotein profile, the uptake/production and lipolysis/production ratios in VLDL can be quantified. The information from these ratios can be summarized in a single statistic taking the mean of these two ratios, which can be visualized as a projection on the identity line. We propose the name VLDL performance for this projection. It integrates information about production, lipolysis and uptake rates, but can be calculated without metabolic flux information, based on one detailed lipoprotein profile measured in one fasting blood sample.

We aim at applying Particle Profiler to lipoprotein profiles and deriving lipoprotein metabolic ratios, as illustrated in Figure 2. As a first step towards this implementation, we here introduce two lipoprotein metabolic ratios, which we calculate in this study by applying Particle Profiler to pooled lipoprotein flux data.

## Lipoprotein metabolic ratios for VLDL metabolism

Since overproduction of large VLDL particles is an important characteristic of the atherogenic lipoprotein phenotype [27], we first examined VLDL metabolism. The only current clinical marker reflecting VLDL is total plasma triglycerides, but this marker also includes triglycerides in chylomicrons, IDL, LDL and HDL. The marker introduced here relates more specifically to VLDL.

Based on Particle Profiler, we derived ratios between hepatic VLDL uptake and production, and between LPL-related VLDL lipolysis and production. Since the model calculates the metabolic rates of VLDL at each particle size, this complex information needs to be integrated into a single value. Therefore, we calculated three ratios of metabolic rates of a VLDL particle. These are $\frac{\bar{k_{u,liver}^{VLDL}}}{J_{p,VLDL}}$, $\frac{\bar{k_{l}^{VLDL}}}{J_{p,VLDL}}$, and VLDL performance, whose mathematical definition can be found below. A schematic introduction can be found in Figure 2. When referring to the 'VLDL metabolism ratios' we refer to these three ratios.

The VLDL uptake - production ratio $\frac{\bar{k_{u,liver}^{VLDL}}}{J_{p,VLDL}}$ can be calculated from modeled values as follows *(eq. 4)*:

$$\frac{\bar{k_{u,liver}^{VLDL}}}{J_{p,VLDL}}=\frac{R+\overset{d_{i,j}\leq d_{b}-\frac{1}{2}d_{i,j}^{r}}{\underset{d_{i,j}\geq d_{a}+\frac{1}{2}d_{i,j}^{r}}{\sum }}k_{u,liver}\left(d_{i,j}\right)\cdot \frac{Q_{ss}\left(d_{i,j}\right)}{Q_{VLDL}}}{J_{p,VLDL}}$$

Where *k_u, liver_(d_i, j_) *is the hepatic uptake rate and *Q_ss_(d_i, j_) *is the concentration of particles in the subclass with average diameter *d_i, j _*. The meaning of subindices *i *and *j *relates to the position of the subclass in the lipolysis cascade, which is explained in [13] under 'lipolysis cascades'. *Q_VLDL _*and *J_p, VLDL _*are the total concentration of VLDL particles and total VLDL production influx respectively, where VLDL covers the size range from *d_a _= 30 nm *to *d_b _= 80 nm*. *R *is the linearly interpolated small remainder for the boundary subclasses, which partially fall in the selected range *(eq.4a)*:

$$R_{low}=\frac{\left(d_{i,j}+\frac{1}{2}d_{i,j}^{r}\right)-d_{a}}{d_{i,j}^{r}}\cdot k_{u,liver}\left(d_{i,j}\right)\cdot \frac{Q_{ss}\left(d_{i,j}\right)}{Q_{out}\left(\left[d_{a},d_{b}\right]\right)}$$

where $d_{a}\in \left[d_{i,j}-\frac{1}{2}d_{i,j}^{r},d_{i,j}+\frac{1}{2}d_{i,j}^{r}\right]$

$$R_{high}=\frac{d_{b}-\left(d_{i,j}-\frac{1}{2}d_{i,j}^{r}\right)}{d_{i,j}^{r}}\cdot k_{u,liver}\left(d_{i,j}\right)\cdot \frac{Q_{ss}\left(d_{i,j}\right)}{Q_{out}\left(\left[d_{a},d_{b}\right]\right)}$$

where $d_{b}\in \left[d_{i,j}-\frac{1}{2}d_{i,j}^{r},d_{i,j}+\frac{1}{2}d_{i,j}^{r}\right]$

$$R=R_{high}+R_{low}$$

Where $d_{i,j}^{r}$ represents the radius of the subclass with average diameter *d_i, j _*.

The ratio between LPL-related lipolysis and production in VLDL ($\frac{\bar{k_{l}^{VLDL}}}{J_{p,VLDL}}$) is defined analogously, by replacing *k_u, liver _*(*d_i, j_*) in equations 4 and 4a by *k_l_(d_i, j_)*.

To show in a single value how efficiently produced VLDL particles are metabolized, we introduce the 'VLDL performance' diagnostic, which is the average of the previous two ratios.

$$VLDLperformance=\frac{\frac{\bar{k_{u,liver}^{VLDL}}}{J_{p,VLDL}}+\frac{\bar{k_{l}^{VLDL}}}{J_{p,VLDL}}}{2}$$

Both ratios and VLDL performance have the dimension volume * particles^-1^. Figure 2 graphically shows how VLDL performance and the two ratios are related, and how different disturbances in VLDL metabolism affect VLDL performance.

## General dyslipidemia

In order to obtain an indication of the clinical relevance of our new markers for VLDL metabolism, we applied the Particle Profiler model to a range of published studies with pooled lipoprotein flux data obtained in dyslipidemic subjects. Studies that contain both pooled lipoprotein flux data and 'hard' endpoints such as cardiovascular events are not available; dyslipidemia is the closest possible alternative. In nearly all the selected studies a group of healthy subjects and a group of patients showing a specific type of dyslipidemia were investigated and compared. Our pool of dyslipidemic subjects was defined as all the subjects that were considered to be 'dislipidemic' in each study. To be included in our analysis the studies also had to report data of standard clinical chemistry for comparison with our new markers. Subjects for which the measured particle influx and efflux differed more than 10% in one class (LDL, IDL, VLDL1 or VLDL2) were judged not to be at steady state and excluded from the dataset. A summary of the data used for the analysis can be found in Table 3. Since all these studies contain lipoprotein flux data, the number of subjects measured is limited. The dyslipidemic state of the selected patients is always very clearly distinguishable from the normolipidemic state, so that an effect can be observed with a small number of patients.

**Table 3:** Subject groups used

Subject group	Number of subjects	**Ref**.	Included in 'normo-lipidemic' group	Included in 'dys-lipidemic' group
Normolipidemic controls	3 (in: N1, N3, N5)	[19]	x	
Normolipidemic controls	6	[20]	x	
Normolipidemic controls: apoE 3/3 subjects.	5	[17]	x	
Normolipidemic controls	9	[21]	x	
All subjects	12	[22]	x	
phenotype 'A' (large LDL particle size)	9	[18]	x	
mixed dyslipidemia prior to treatment (Baseline)	5 (in: P2, P3, P5, P7, P8)	[24]		x
mixed dyslipidemia prior to treatment (Baseline)	11	[25]		x
kidney patients	9	[21]		x
hypothyroid subjects before and during T4 therapy	10 (excluded before T4: 6; during T4: 3)	[26]		x
HIV treatment-associated hyperlipidemic subjects	5	[23]		x
phenotype 'B' subjects, with small LDL particle size	4 (in: subjects 17-20)	[18]		x
LPL -/-	3	[16]		
apoE 2/2	4	[17]		
apoE 4/4	5	[17]		
homozygous familial hypercholesterolemia	3 (in: FH1, FH2, FH4)	[19]		
familial defective apoB	3	[29]		
S447X; a single nucleotide polymorphism in the LPL gene	5	[30]		
Total used for normolipidemic group	44			
Total used for dyslipidemic group	44			

Subject groups used for normolipidemia, dyslipidemia, and genetic disorders. If subjects needed to be excluded from a group because of a lack of steady state in the data (in- and efflux balance), individual subjects are mentioned.

We tested whether particle Profiler-derived VLDL performance was different for normolipidemic versus dyslipidemic subjects. This test also examined the difference in VLDL performance in relation to differences in standard diagnostics: triglycerides, HDL cholesterol, and LDL cholesterol. The parameters we introduce are completely novel; there is no similar diagnostic method that we can compare them to. The differences with standard diagnostics were expressed as the ability to correctly predict the known normolipidemic or dyslipidemic state from the diagnostic parameters. The test consisted of two phases: first the predictive power of each diagnostic separately, then the predictive power of multivariate models. For the multivariate models we performed logistic regression, we subsequently added LDL cholesterol, HDL cholesterol, triglycerides and VLDL performance as predictor variables. The diagnostic accuracy was quantified using ROC curves [28]. We used both the Area Under the Curve (AUC) and Partial Area Under the Curve statistics (pAUC - for false positive rates < 0.2) [28] to quantify the predictive power of each separate diagnostic and of each multivariate regression model.

**Figure 3:**
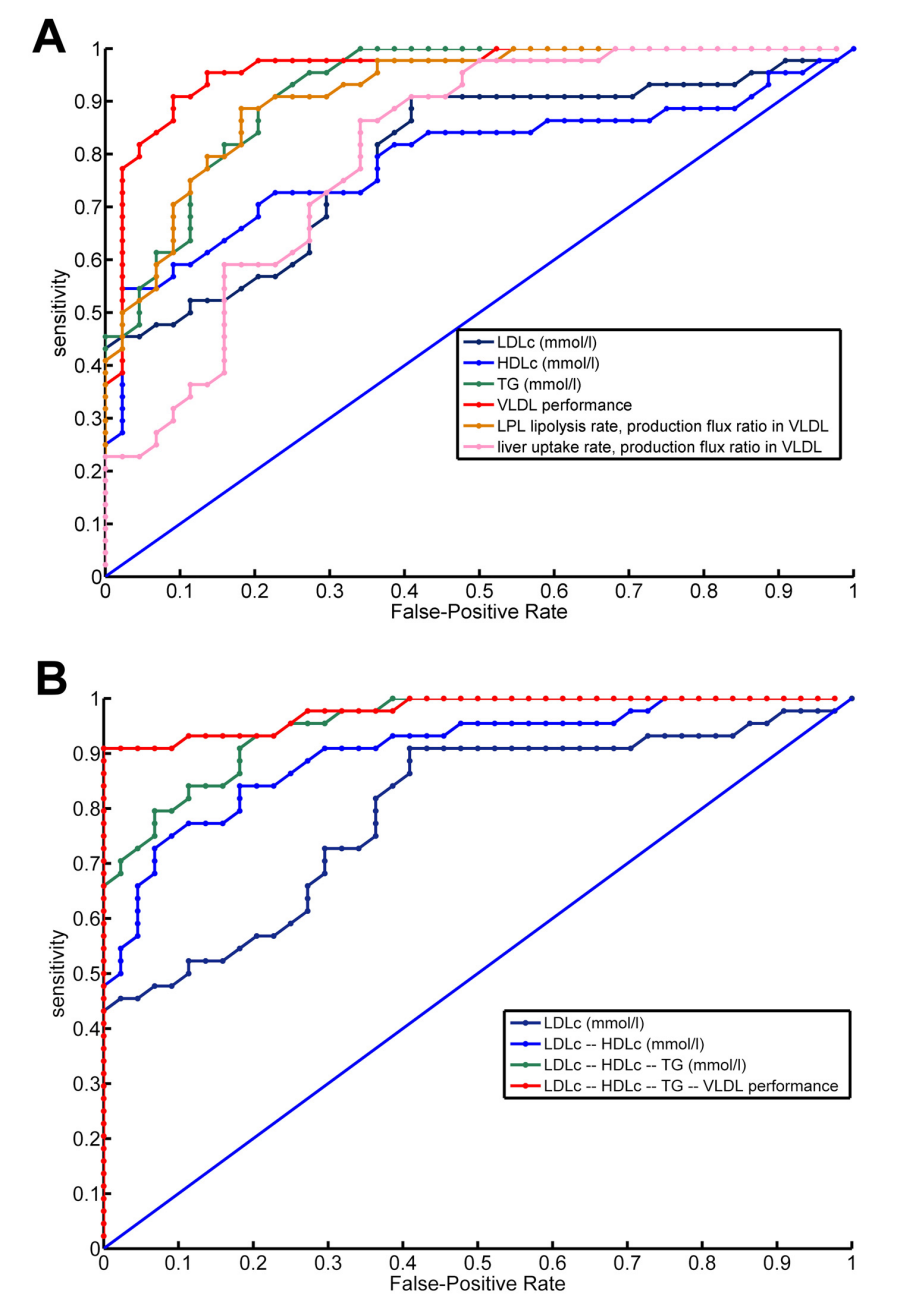
**Receiver operating characteristic (ROC) curves for dyslipidemia**. These curves indicate how well a) single diagnostics and b) multivariate regression models distinguish dyslipidemic subjects from normolipidemic subjects. The models in b) subsequently include LDLc, HDLc, TG, and VLDL performance in cumulative fashion. The ROC curve indicates with what sensitivity various diagnostics can identify dyslipidemic subjects, when varying the acceptable false-positive rate. An ROC curve further away from the 1-1 identity line indicates a better diagnostic. For example, when not accepting false positives, the regression model including LDLc, HDLc and TG has a sensitivity of 66% (0.66), while additionally including the VLDL performance diagnostic results in a sensitivity of 91% (0.91).

The new VLDL performance marker clearly differed between normolipidemic and dyslipidemic subjects. Figure 3a shows ROC curves that describe how well LDL cholesterol, HDL cholesterol, triglycerides, and VLDL performance distinguished normolipidemic from dyslipidemic subjects. Figure 3b shows ROC curves of multivariate regression models that successively incorporate these diagnostics for making the same distinction together. Table 4 shows the partial area-under-the-curve (pAUC) and area-under-the-curve AUC values, indicating diagnostic power for dyslipidemia, for each of the regression models. The table shows that VLDL performance distinguished normolipidemics better from dyslipidemics than the routine clinical chemistry parameters. Also, VLDL performance improved the distinction when used in combination with LDL cholesterol, HDL cholesterol and triglycerides. When accepting no false positives, the model including VLDL performance had a 91% sensitivity for correctly identifying dyslipidemic subjects, versus a 67% sensitivity when using only triglycerides, HDLc and LDLc. Therefore, VLDL performance had additional value for distinguishing dyslipidemic from normolipidemic subjects compared to standard diagnostics.

**Table 4:** Power of distinction between normolipidemic and dyslipidemic subjects

Rank	Diagnostic	pAUC	AUC
1	LDLc -- HDLc -- TG -- VLDL performance	0.184	0.955
2	VLDL performance	0.167	0.937
3	LDLc -- HDLc -- TG	0.159	0.929
4	LDLc -- HDLc	0.141	0.881
5	$\frac{\bar{k_{l}^{VLDL}}}{J_{p,VLDL}}$	0.133	0.893
6	TG (mmol/l)	0.130	0.900
7	HDLc (mmol/l)	0.112	0.790
8	LDLc (mmol/l)	0.099	0.794
9	$\frac{\bar{k_{u,liver}^{VLDL}}}{J_{p,VLDL}}$	0.071	0.783

Partial area under the curve (pAUC) and area under the curve (AUC) calculated from ROC curves of various diagnostics and combinations of diagnostics for distinguishing dyslipidemic subjects from normolipidemic subjects. Both pAUC and AUC are a measure of how well each diagnostic predicts the dyslipidemic status, with the difference that the pAUC only takes into account those predictions for which the false positive rate is smaller than 0.2, while the AUC takes into account all possible false positive rates. The higher the pAUC and AUC are, the better the diagnostic is.

## Dyslipidemic subgroups

Next, we examined the average value of our VLDL-related diagnostic parameters for each of the studied subgroups. This comparison included subject groups for which no standard clinical chemistry parameters were available (normolipidemic subjects from [17-22]) and subject groups with specific genetic disorders. These genetic disorders include LPL -/- [16], apoE 2/2 [17], apoE 4/4 [17], homozygous familial hypercholesterolemia [19], homozygous familial defective apoB [29], and S447X [30] - a single nucleotide polymorphism in the LPL gene.

**Figure 4:**
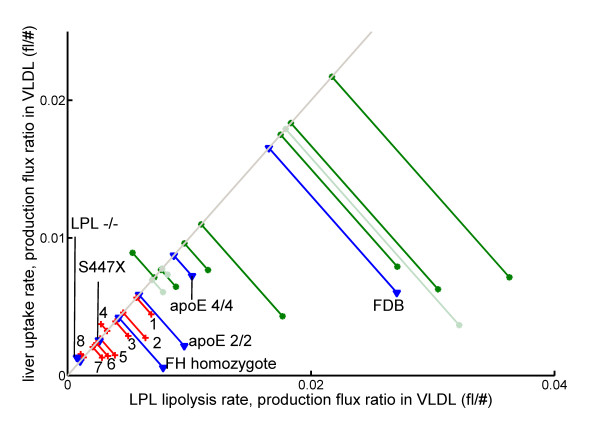
**The average VLDL performance of various subject groups**. Green lines with round ends are normolipidemic subject groups. Groups indicated with darker green were used for the ROC curve in figure 3, those indicated with light green were not. Red lines with crosses represent dyslipidemic subject groups used for the ROC curve. Groups are labeled as follows: 1) hypothyroid patients during T4 treatment [26]; 2) subjects with small LDL peak size [18]; 3 and 8) mixed hyperlipidemia [24,25]; 4) hypothyroid patients before treatment [26]; 5) kidney disease: membranous glomerulonephritis [21]; 6) patients on HIV treatment [23]; 7) kidney disease: focal segmental glomerulosclerosis [21]. Blue lines with triangles indicate subject groups with specific genetic variant. FDB: Familial Defective ApoB (mostly heterozygote) [29]; FH: Familial Hypercholesterolemia (homozygote) [19]; S447X: specific single nucleotide polymorphism in the LPL gene [30].

Figure 4 shows the average VLDL metabolism-ratios for all included subject groups. The figure clearly indicates that the normolipidemic groups (green and light-green lines) had a higher VLDL performance (projection onto the identity line) than dyslipidemic groups. Interestingly, this mainly seems to be due to an increased LPL lipolysis - production ratio in VLDL, although the liver uptake - production ratio in VLDL is generally also higher. In the dyslipidemic patients the VLDL ratios were lower to a different extent. Hypothyroid patients on T4 treatment (marked with '1') seemed to have an improved VLDL performance with respect to the untreated patients (marked with '4'). Patients with small LDL particles showed a relatively light dyslipidemia, whereas mixed hyperlipidemia was associated with different degrees of dyslipidemia ('3' and '8'). Kidney patients, hypothyroid patients, and HIV-treated patients fell in between the mixed hyperlipidemias. The patients with genetic deficiencies showed up at the expected locations. LPL deficient patients had the lowest VLDL performance, and also the S447X polymorphism in this gene negatively affected VLDL performance. ApoE 4/4 subjects, that are known to display a good VLDL clearance, showed up together with the normolipidemics, whereas apoE 2/2 subjects, known to have impaired VLDL clearance, showed up as slightly dyslipidemic.

## Response to treatment

A diagnostic's clinical usefulness increases if there are treatment options when the diagnostic indicates illness. Therefore, we investigated how our VLDL-related diagnostic parameters respond to treatment. For this purpose we used data on atorvastatin and fenofibrate treatment in mixed dyslipidemic subjects from Bilz *et al*. [24], and atorvastatin and simvastatin treatment in mixed dyslipidemic subjects from Forster *et al*. [25]. All these studies contain lipoprotein kinetics data at baseline and after each treatment, which we used as input for Particle Profiler.

**Figure 5:**
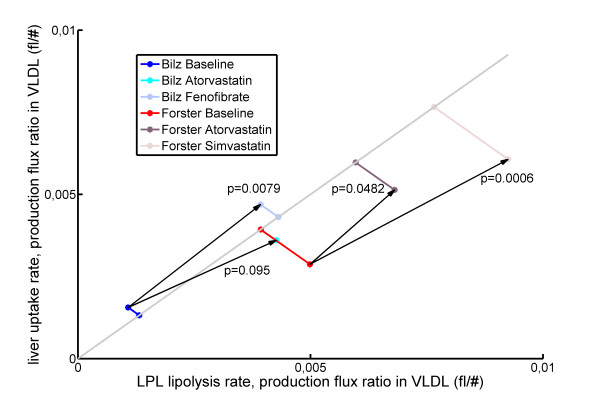
**VLDL performance response to treatment**. Average VLDL performance response (on identity line) to Atorvastatin, Simvastatin and Fenofibrate treatment in mixed hyperlipidemic patients [24,25]. P-values for VLDL performance were calculated by the Wilcoxon rank-sum test. Bilz Atorvastatin: n = 5, p = 0.0925; Bilz Fenofibrate: n = 5, p = 0.0079; Forster Atorvastatin: n = 9, p = 0.0482; Forster Simvastatin: n = 11, p = 0.0006. All treatments caused VLDL performance to move towards healthier values.

Figure 5 shows how the VLDL metabolism diagnostics responded to treatment in mixed dyslipidemic patients. Fenofibrate and simvastatin clearly raised VLDL performance values. The effect of atorvastatin was borderline significant in the study by Forster *et al*. [25], while the study by Bilz *et al*. [24] had too few subjects to distinguish this effect. Therefore, we conclude that fenofibrate and simvastatin have a stronger effect on VLDL performance than atorvastatin.

## Discussion

This study concerns the further development, calibration, and technical and clinical validation of the computational model Particle Profiler. Since no direct measurements can be done for this validation, we investigated whether the model could be corroborated and whether model-derived metabolic ratios for VLDL were able to indicate relevant differences between normolipidemic and dyslipidemic subjects.

### Model development and calibration

Model development involved introducing altered mathematical functions to represent hepatic lipolysis and uptake. The new model implementation had one less free model parameter than the original implementation. The newly fixed parameter contains the particle size at which hepatic lipase (HL) activity is maximal. In continuation, for calibrating the model constants, we used published data from LPL deficient, apoE 3/3 and apoE 2/2 subjects. The values that were determined for the model constants indicate that HL mainly affects smaller VLDL 2 and IDL particles, whereas LPL mainly affects the larger VLDL 2 and VLDL 1 particles. This result corresponds to the known activity of these enzymes [10,16,31].

The calibrated model produced an analysis of lipoprotein kinetics data from Packard *et al*. [18], in which the overall error was comparable to that found in the first model implementation [13], while the new implementation had a smaller standard deviation of the error. It is encouraging that with fewer free parameters the newly optimized model had an approximately equal fit error. The smaller standard deviation of the fit error indicates that the three subjects for which the uptake flux was not fitted well in the initial implementation were fitted better in the current implementation.

In our first paper, we compared the modeled LDL peak size between groups with measured differences in LDL peak size [13]. As could be expected, modeling the HL lipolysis with one less free parameter affected the modeled difference of LDL peak size between groups, because HL is mainly responsible for remodeling of LDL. However, we still detect an LDL peak size difference between groups, although with less significance than before. Closer inspection showed that the new model indeed predicted less difference in HL lipolysis between groups than before, again reflecting the less refined representation of HL in the new model implementation. Conversely, we detected clearer differences in LPL lipolysis. This last change is likely to be a consequence of the model calibration performed with data from genetically deficient subjects. This calibration allowed a clearer distinction of which lipolysis process is operating at what particle size. Therefore, the new model implementation has become somewhat less suitable to identify changes in the metabolism of LDL particles, but has gained power for analyzing the metabolism of IDL and VLDL particles. Overall, we have carried out a necessary simplification of the model, while keeping the model performance stable.

### Model corroboration

The only available option for the technical model validation was corroborating the model, since the model outcomes cannot be measured directly. For this corroboration, we applied the model to lipoprotein kinetics data measured in a range of normolipidemic and dyslipidemic subjects. The model corroboration showed that Particle Profiler was able to fit most lipoprotein kinetics data with an error of 8.8% ± 5.0%, disregarding a study [21] where VLDL2 levels were abnormally high compared to other literature. In conclusion, Particle Profiler is able to accurately analyze lipoprotein kinetics data from subjects with a wide range of dyslipidemias. This corroborates the validity of using the model to analyze lipoprotein flux data.

### VLDL metabolic ratios

We studied VLDL metabolic ratios derived from Particle Profiler. The VLDL performance diagnostic was able to distinguish normolipidemic from dyslipidemic subjects. It did so better than a multivariate regression model including LDLc, HDLc and triglycerides, with a 5% improved pAUC when used alone, and a 16% improved pAUC when added to the multivariate model. These results show that VLDL performance distinguished between normolipidemic and dyslipidemic subjects more clearly than standard clinical chemistry parameters.

In normolipidemics, the ratio between LPL lipolysis and production in VLDL was generally higher than the ratio between liver uptake and production in VLDL. Since the normal physiological function of lipoproteins is to transport triglycerides from the liver to other tissues, it seems perfectly reasonable that extrahepatic lipolysis of VLDL is more important than direct liver uptake in normolipidemic subjects. Subjects with genetic deficiencies showed up at the expected places in the between-group comparison. LPL deficiency or impairment greatly decreased VLDL performance, apoE 2/2 subjects had a slightly less impaired VLDL performance, and apoE 4/4 subjects had a healthy VLDL performance, all according to expectation. The FH patients showed a lower VLDL performance then the FDB subjects, probably because most FDB subjects were heterozygous and the FH subjects homozygous. Treatment by atorvastatin, simvastatin, and fenofibrate all positively influenced the VLDL metabolism ratios, although atorvastatin did so less clearly than the other two treatments. This result is coherent with the findings in the original studies [24,25] that all drugs increased VLDL turnover rates; also, in both these studies atorvastatin affected the VLDL1 turnover less strongly than either fenofibrate or simvastatin. Taken together, the results mentioned above show that the VLDL metabolism ratios clearly reflect dyslipidemic status, and that drug therapy improves dyslipidemia as quantified by these ratios. These results constitute a first indication of clinical usefulness of lipoprotein metabolism ratios based on Particle Profiler.

### Future development

We plan to conduct a further clinical validation of Particle Profiler-based metabolic ratios as predictors of cardiovascular disease in a separate study. To this end we will analyze relevant data from cohorts such as the Framingham Heart Study. Developing a similar approach to Particle Profiler for HDL metabolism is also a future possiblity. Since VLDL metabolism is known to be affected in the 'atherogenic lipoprotein phenotype' [27], there is reason to believe that parameters derived from the current Particle Profiler implementation can contribute to predicting cardiovascular disease risk. Parties interested in working with Particle Profiler are requested to contact TNO.

## Conclusions

In this study we further developed, calibrated, and corroborated the Particle Profiler computational model using pooled lipoprotein metabolic flux data. From pooled lipoprotein metabolic flux data on dyslipidemic patients, we derived VLDL metabolic ratios that better distinguished normolipidemic from dyslipidemic subjects than standard diagnostics (HDLc, TG, LDLc). Since dyslipidemias are closely linked to cardiovascular disease and diabetes type II development, lipoprotein metabolic ratios are candidate risk markers for these diseases. These ratios can in principle be obtained by applying Particle Profiler to a single lipoprotein profile measurement, which makes clinical application feasible.

## Methods

### Model Parametrisation: Overview

The model equations have been parameterized as follows:

**Production: **no fitted parameters (eq. 1&2 in [13])

The *J_in _*'s are based directly on the dataset being fitted, other values can be found in appendix 1.

**Extrahepatic lipolysis: **1 fitted parameter (eq. 4 in [13])

*k_l, max _*maximum rate at which extrahepatic lipolysis takes place

2 fixed model constants:

*d_l, min _*minimum size at which extrahepatic lipolysis occurs

*σ_l _*shape parameter for extrahepatic lipolysis

**Liver attachment, lipolysis and uptake: **5 fitted parameters (eqs. 1-3)

*k_a, apoEmax _*maximum rate at which liver binding mediated by apoE takes place

*k_a, apoB _*rate at which liver binding mediated by apoB takes place

*A *shape constant for liver binding mediated by apoE

*B *shape parameter for liver binding mediated by apoE

*σ_u, liver _*shape parameter describing how the fraction of liver attachment which is taken up (instead of lipolized) varies with particle size.

2 fixed model constants

*S_u, liver _*shape constant describing how the fraction of liver attachment which is taken up (instead of lipolized) varies with particle size.

*d_l, apoEmin _*minimum particle diameter at which liver binding mediated by apoE takes place

**Triglyceride loss during lipolysis **(eq 8 in [13])

1 fixed model constant

*f_tg _*fraction of triglycerides lost at each lipolysis step

### Model reparameterisation for fitting of flux data

Using the parameters specified above, the fitting routine had difficulty to find the global minimum mean square error. In order to improve its performance we specified a reparametrisation, specific for the data type used in this paper. This allows the fitting routine to find a minimum without difficulty.

*k_l, max _*maximum rate at which extrahepatic lipolysis takes place

(unchanged from original model)

*B *shape parameter for liver binding mediated by apoE.

(unchanged from original model)

*σ_u, liver _*shape parameter describing how the fraction of liver attachment which is taken up (instead of lipolized) varies with particle size.

(unchanged from original model)

*k_a, apoB _*the liver attachment rate due to apoB-related processes

(unchanged from original model)

*k*_*peak *_the total rate of all processes at the particle size at which hepatic lipolysis is at its peak (*d = d_hl, peak_*).

Fixed model constant

*d_hl, peak _*Size at which hepatic lipolysis is at its peak

The new parameter is specified as follows:

(5) $$k_{peak}=\;k_{a,liver}\left(d_{hl,peak}\right)\;+\;k_{l}\left(d_{hl,peak}\right)$$

This specification of parameters may lead to problems in the fitting routine in specific cases, when the boundary values of processes are reached. Therefore, during the parameter conversion process from fitted parameters to parameters for model calculation, the following fitted parameters were dynamically set to their boundary value. This procedure means that a parameter is only set to its boundary value if necessary, otherwise it is fitted normally.

Condition: *k*_peak _*≤ k*_a, apoB _+ *k*_*l*, max_

Set boundary: *k*_peak _*= k*_a, apoB _+ *k*_*l*, max _+0.00001

Condition: *σ_u, liver _*> 25

Set boundary: *σ_u, liver _*= 25

Condition: B<1+elnd-da,liver minsu,liver1∕2⋅σu,liver⋅su,liver⋅su,liver

Set boundary: B=1+elnd-da,liver minsu,liver1∕2⋅σu,liver⋅su,liver⋅su,liver+0.000001

### Size classes

The size range of each size class are shown in Table 5.

**Table 5:** Size Classes

Subfraction	Minimum size	Maximum size
LDL	5	25.0
IDL	25.0	30.0
VLDL2	30.0	36.0
VLDL1	36.0	60

The size range of each size class has been estimated as shown in this table, modified from [32].

### Error function

The error function used in the nlinfit routine is shown in Table 6. The lower weights of the larger particle pools give them more importance in the fitting routine. This is desirable since these pools are also important to estimate the rate of the fluxes correctly.

**Table 6:** Error function

	LDL	IDL	VLDL 2	VLDL 1
Particle pool scale factor (particles/fl)	3	2	2	1
Lipolysis efflux scale factor (min^-1^)	-	0.005	0.005	0.005
Uptake flux scale factor (min^-1^)	0.005	0.005	0.005	0.005

The nlinfit routine calculates a sum of square difference between data points and model predictions. Before entering into this routine the data was scaled a) to correct for different units of pools data and flux data and b) to indicate the relative importance of each data point. This adjustment is specific for the type of data used. Data and model predictions were divided by the scaling factors shown in this table.

To indicate the error value between the data and the model fit a percentage error was defined. This score was not used for model fitting, only for reporting an intuitive error score. It takes into account the number of data points for pools, uptake and lipolysis and the higher importance (double of the flux total) of the pool sizes; they are most important for parameter estimation. It is given by the following formula *(eq. 6)*:

$$E=\left\{\begin{array}{c} \frac{16}{23}\cdot \frac{\underset{i}{\sum }|Q_{i}^{d}-Q_{i}^{m}|}{\underset{i}{\sum }Q_{i}^{d}}+\\ +\frac{3}{23}\frac{\underset{i}{\sum }|J_{i}^{l,d}-J_{{}_{i}}^{l,m}|}{\underset{i}{\sum }J_{i}^{l,d}}+\frac{4}{23}\frac{\underset{i}{\sum }|J_{i}^{u,d}-J_{{}_{i}}^{u,m}|}{\underset{i}{\sum }J_{i}^{u,d}} \end{array}\right)$$

Where E stands for error value, Q indicates the pool size, superscript d indicating the data and m the model fit. J stands for a flux, superscripts d and m as before, l indicates lipolysis, u indicates uptake. Subscript i indexes the different data points of each lipolysis size class, i.e. LDL, IDL, VLDL2 and VLDL1.

### Conversions

The datasets of Packard *et al*. [18], Demant *et al*. [16,17], and Bilz *et al*.[24] contain estimations for the lipoprotein pools of the various classes in mg, and turnover speeds in pools per day. These are converted to particle concentrations and particle fluxes respectively. This needs the assumption that only ApoB-100 is present on lipoprotein particles in the fasted state, which is reasonable given that apoB-48 is produced by the intestine mainly postprandially. The equation looks as follows (*eq. 7*):

$$n\left(\frac{mol}{L}\right)=\frac{n\left(g\right)}{M_{ApoB-100}\left(g∕mol\right)\cdot V_{blood}\left(L\right)}$$

Where n is the number of lipoproteins, M_ApoB-100 _the molar mass of ApoB-100 and V_blood _the blood volume of an individual person (taken to be 5 L).

## Competing interests

DBvS and APF are named inventors on a patent application for the Particle Profiler model, owned by TNO quality of life and the University of Leiden. DBvS and AAdG are named inventors on a patent application for Particle Profiler-derived markers for cardiovascular disease (based on follow-up of the present work), owned by TNO.

## Authors' contributions

DBvS did the modeling work and drafted the manuscript. AAdG participated in the design of the study and helped to draft the manuscript. BvO, AF, and JvdG conceived of the study, participated in its design, and coordination and helped to draft the manuscript. All authors read and approved of the final manuscript.

## Endnotes

^1 ^article freely downloadable from http://www.jlr.org/content/50/12/2398

## Supplementary Material

Additional file 1 **Motivation for the equations**. A complete, step-by-step motivation for the new equations introduced in this study. Click here for file
