# Supplementary material for: Diagnostic markers based on a computational model of lipoprotein metabolism
Source: J Clin Bioinforma. 2011 Oct 26;1:29. doi: 10.1186/2043-9113-1-29 (PMC3305892; doi:10.1186/2043-9113-1-29)

Diagnostic Markers based on a Computational Model of Lipoprotein Metabolism

Daniël B. van Schalkwijk[1,2,3,§], Ben van Ommen[1], Andreas P. Freidig[4], Jan van der Greef[1,2] Albert A. de Graaf[1]

[1] TNO Quality of Life, Business Unit Biosciences, Zeist and Leiden, the Netherlands

[2] Leiden Amsterdam Centre for Drug Research (LACDR), Analytical Sciences division, Leiden, the Netherlands

[3] The Netherlands Bioinformatics Centre (NBIC), Nijmegen, the Netherlands.

[4] Amsterdam Molecular Therapeutics (AMT), Amsterdam, the Netherlands

[§]Corresponding author

Email addresses:

DBvS: daan.vanschalkwijk@tno.nl

BvO: ben.vanommen@tno.nl

APF: a.freidig@amtbiopharma.com

JvdG: jan.vandergreef@tno.nl

AAdG: albert.degraaf@tno.nl

Additional Material

Motivation for the equations

The Particle Profiler model describes how lipoprotein production, lipolysis and uptake processes depend on lipoprotein size. Equations for this size dependence cannot be derived mechanistically, since biological knowledge does not suffice for such a derivation. Biological knowledge does, however, provide clues as to how these functions should look. These clues are mentioned in the 'biology' paragraph of the first article describing the model [13][1] and their interpretation is mentioned in the 'conceptual model' paragraph of each modeled process in the same article. Based on this information we can motivate the Particle Profiler equations. How this motivation translates into the Particle Profiler equations is explained in the additional material of the first article [13]. We here provide a similar motivation for the new equations.

Liver - lipolysis and uptake

Liver attachment

In our model particles can be processed by the liver in two ways. They can be directly taken up via an apoB-related mechanism. They can also be attached to the liver via an apoE-related mechanism, which can result either in particle uptake or lipolysis. The apoB-related uptake mechanism is modeled to have the same rate at all particle sizes, which therefore always equals $k_{u,apoB}$.

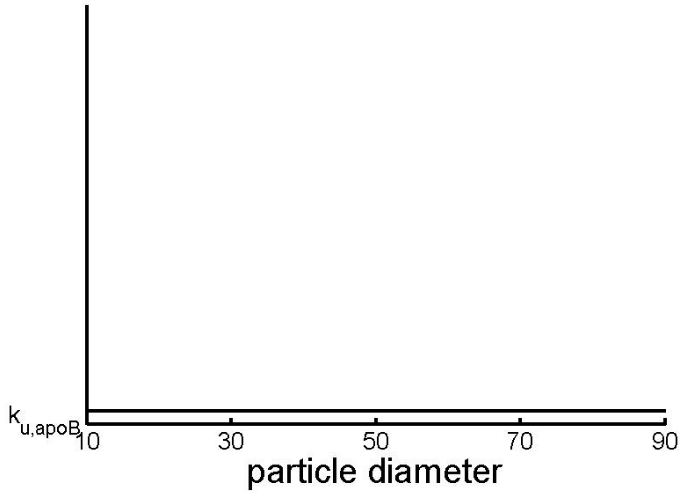

For apoE-related attachment to the liver we expect a pattern which first increases and then decreases with particle size. Furthermore, the initial increase should be gradual, so that the liver can gradually increase the lipolysis of smaller particles. The probability density function of the single parameter Rayleigh distribution we initially used, meets the first requirement, but not the second. Therefore, we now switched to the more general Weibull distribution, and constructed suitable one-parameter version of this distribution. This Weibull distribution is not interpreted as a probability distribution, but it rather describes the apoE related attachment rate at different particle sizes as a fraction ($f_{a,apoE}(d)$) of the maximum apoE related attachment rate. This leads to the following first candidate model:

$$f^{1}_{a,apoE}(d) = BA^{-B}d^{B-1}e^{-\left(\frac{d}{A}\right)^{B}} \qquad \textit{eq. 1}$$

---

[1] article freely downloadable from http://www.jlr.org/content/50/12/2398

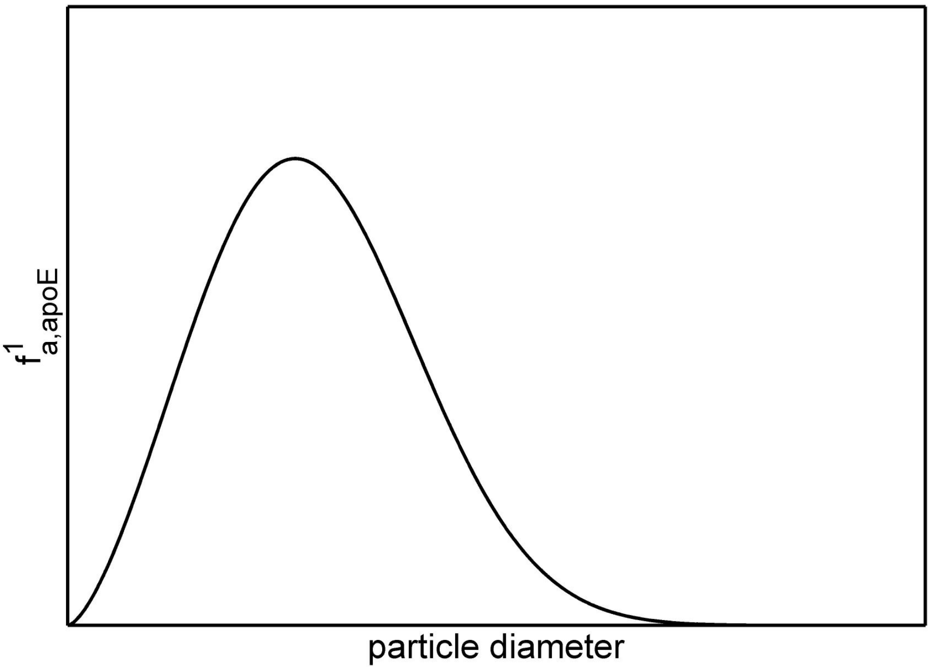

One problem of the equation in this form is that its maximum value does not equal one. This is undesirable since we would like to introduce a parameter which represents the maximum liver attachment rate. This maximum rate should be reached at the peak of the modeled distribution. Therefore we now first scale the Weibull probability density function with its maximum value, which can vary according to the parameter values. We can calculate the value of *d* at which this maximum is reached by equating the derivative of $f^1_{a,apoE}$ to zero. We find that:

$$\frac{d}{dd} f^1_{a,apoE}\left(e^{\frac{\ln\left(\frac{B-1}{B}\right)}{B}}\cdot A\right)=0 \qquad \textit{eq. 2}$$

By scaling the weibull distribution with its maximum, its peak value always equals one. The second candidate model thus becomes:

$$f^2_{a,apoE}(d)=\left(\frac{f^1_{a,apoE}(d)}{f^1_{a,apoE}\left(e^{\frac{\ln\left(\frac{B-1}{B}\right)}{B}}\cdot A\right)}\right)=d^{B-1}\frac{e^{-\left(\frac{d}{A}\right)^{B}}}{e^{\left(\ln\left(\frac{B-1}{B}\right)-1\right)\left(\frac{B-1}{B}\right)}\cdot A^{B-1}} \qquad \textit{eq. 3}$$

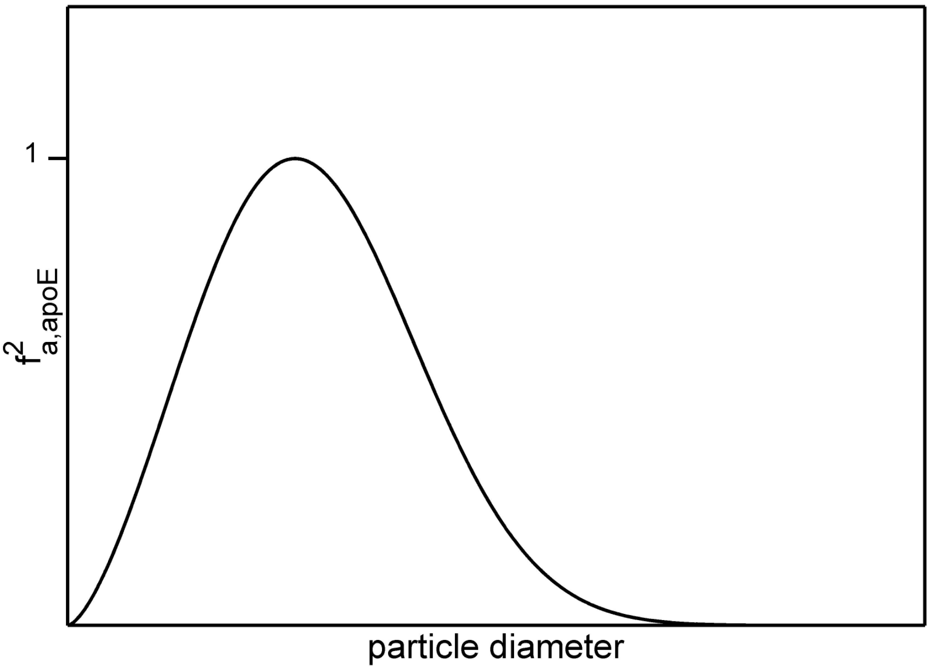

Next, the equation needs to be shifted horizontally to start at the smallest particle size at which liver attachment occurs. The final model for the fraction of maximum apoE-related uptake thus becomes:

$$f_{a,apoE}(d) = \begin{cases} (d - d_{a,apoE\min})^{B-1} \dfrac{e^{\frac{-\cdot(d-d_{a,apoE\min})^B}{A}}}{e^{\left(\ln\left(\frac{B-1}{B}\right)-1\right)\left(\frac{B-1}{B}\right)} \cdot A^{B-1}} & for \quad d \geq d_{a,apoE\min} \\ 0 & for \quad d < d_{a,apoE\min} \end{cases} \qquad eq.\ 4$$

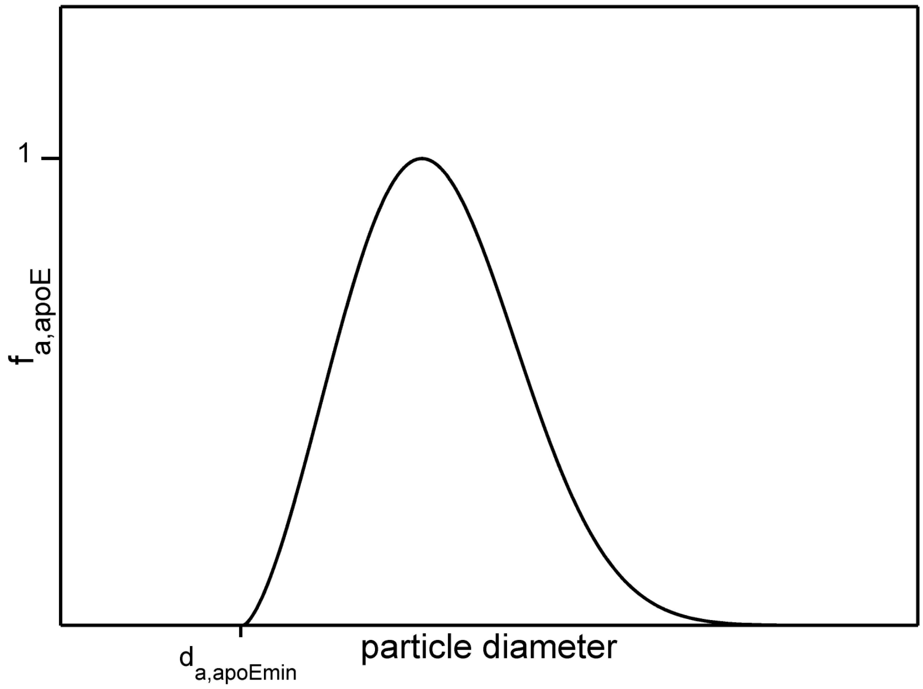

we can now calculate the apoE related attachment rate, by introducing a parameter representing the maximum liver attachment rate of the apoE-related process.

$$k_{a,apoE}(d)=\begin{cases} k_{a,apoE\max}\left((d-d_{a,apoE\min})^{B-1}\dfrac{e^{\frac{-(d-d_{a,apoE\min})^{B}}{A}}}{e^{\left(\ln\left(\frac{B-1}{B}\right)-1\right)\left(\frac{B-1}{B}\right)}\cdot A^{B-1}}\right) & for \quad d\geq d_{a,apoE\min} \\ 0 & for \quad d<d_{a,apoE\min}\end{cases} \quad eq.5$$

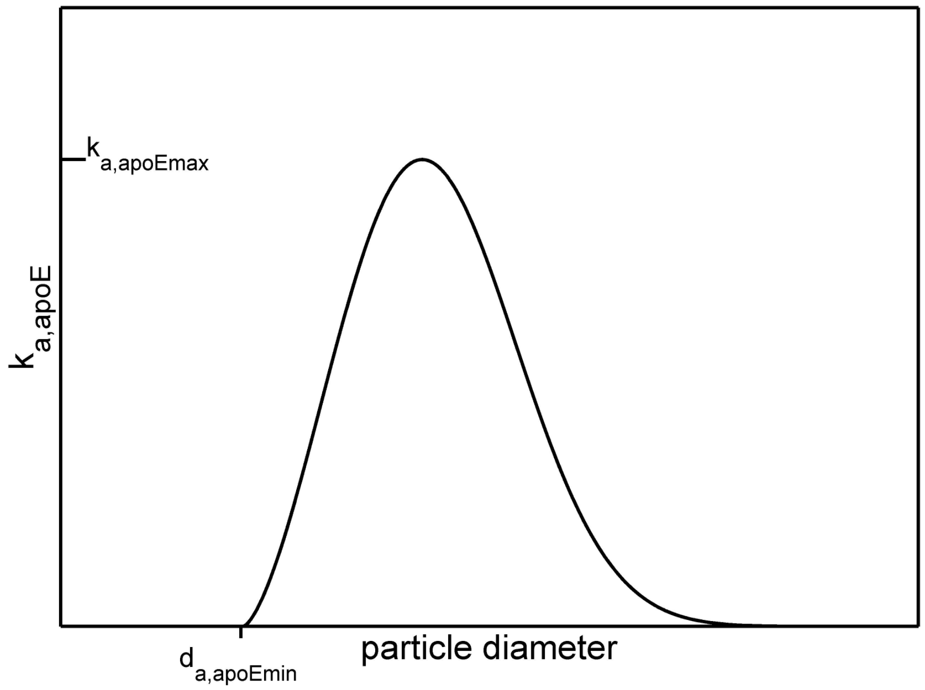

Since liver attachment includes an apoB-related and an apoE-related contribution, we calculate the total liver attachment rate as follows (*eq. 6*):

$$k_{a,liver}(d)=\begin{cases} k_{a,apoE\max}\left((d-d_{a,apoE\min})^{B-1}\dfrac{e^{\frac{-(d-d_{a,apoE\min})^{B}}{A}}}{e^{\left(\ln\left(\frac{B-1}{B}\right)-1\right)\left(\frac{B-1}{B}\right)}\cdot A^{B-1}}\right)+k_{a,apoB} & for \quad d\geq d_{a,apoE\min} \\ k_{a,apoB} & for \quad d<d_{a,apoE\min}\end{cases}$$

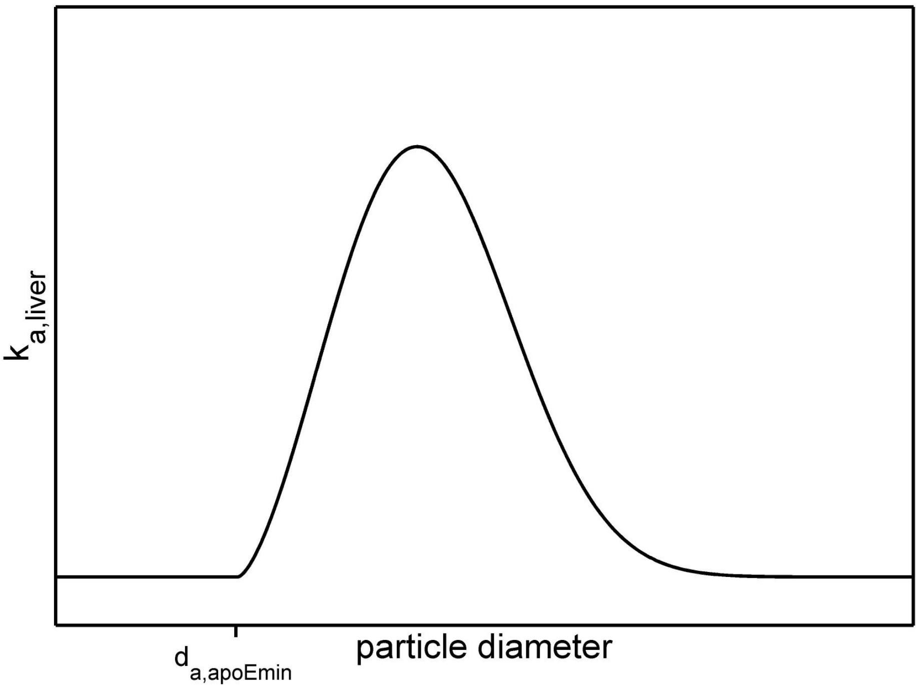

Liver lipolysis and uptake

In the apoB-related mechanism liver attachment is immediately followed by uptake. The apoE-related attachment mechanism can either be followed by lipolysis or uptake. The model for this last process does not include a pool of attached particles. The model rather considers that attachment is directly followed by either uptake or lipolysis. This is obviously a simplification, but a necessary one since data are not available to parameterize a more detailed process. Because of this simplification we can state in terms of fluxes (*J*) that:

$$J_{a,liver}(d) = J_{l,liver}(d) + J_{u,liver}(d) \qquad \textit{eq. 7}$$

which can be rewritten in terms of pools (*Q –dimension: # of particles*) and rate constants (*k – dimension: 1/t*) to the following:

$$Q(d)\cdot k_{a,liver}(d) = Q(d)\cdot k_{l,liver}(d) + Q(d)\cdot k_{u,liver}(d) \qquad \textit{eq. 8}$$

dividing away the pools we get:

$$k_{a,liver}(d) = k_{l,liver}(d) + k_{u,liver}(d) \qquad \textit{eq. 9}$$

In order to model how the ratio between uptake and lipolysis in the liver depends on particle size, we use the consideration that hepatic lipase mainly acts on smaller particles. This means that the fraction of particles taken up after liver attachment increases with increasing particle size. To model this process we use the cumulative probability density function of the Weibull distribution, leading to the following equation for the fraction of attached particles that is taken up by the liver instead of lipolised ($f_{a\rightarrow u,liver}$):

$$f_{a \to u,liver}(d) = \begin{cases} 1 - e^{-s_{u,liver}\frac{1}{2}\left(\frac{d - d_{a,apoE\min}}{\sigma_{u,liver}}\right)} & for \quad d \geq d_{a,apoE\min} \\ 0 & for \quad d < d_{a,apoE\min} \end{cases} \qquad eq.10$$

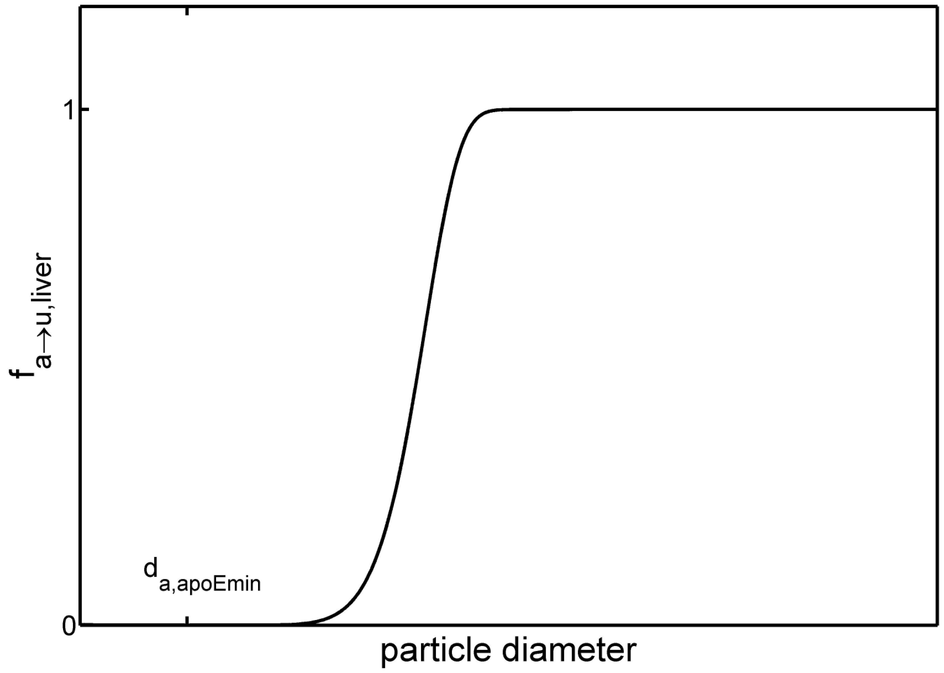

Now, we also need to take into account that in our model hepatic uptake consists of two contributions. The first is that of apoB-related uptake, which is constant irrespective of particle size. The second is that of apoE-related uptake, which does depend on particle size. In the final equation for liver uptake we combine the contributions of both processes:

$$k_{u,liver}(d)=\begin{cases}\left(k_{a,liver}-k_{a,apoB}\right)\left(1-e^{-s_{u,liver}\frac{1}{2}\left(\frac{d-d_{a,apoEmin}}{\sigma_{u,liver}}\right)}\right)+k_{a,apoB} & for \;\; d\geq d_{a,apoEmin}\\ k_{a,apoB} & for \;\; d<d_{a,apoEmin}\end{cases} \quad eq11$$

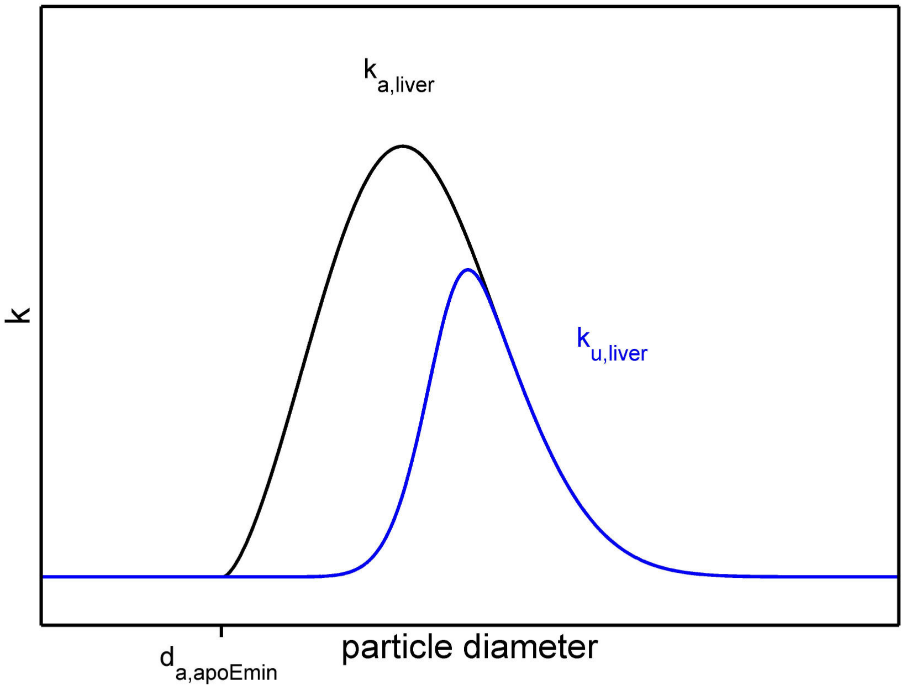

using the relation between hepatic attachment, hepatic uptake and hepatic lipolysis motivated above, we can now calculate hepatic lipolysis as follows:

$$k_{l,liver}(d)=k_{a,liver}(d)-k_{u,liver}(d) \qquad eq.12$$

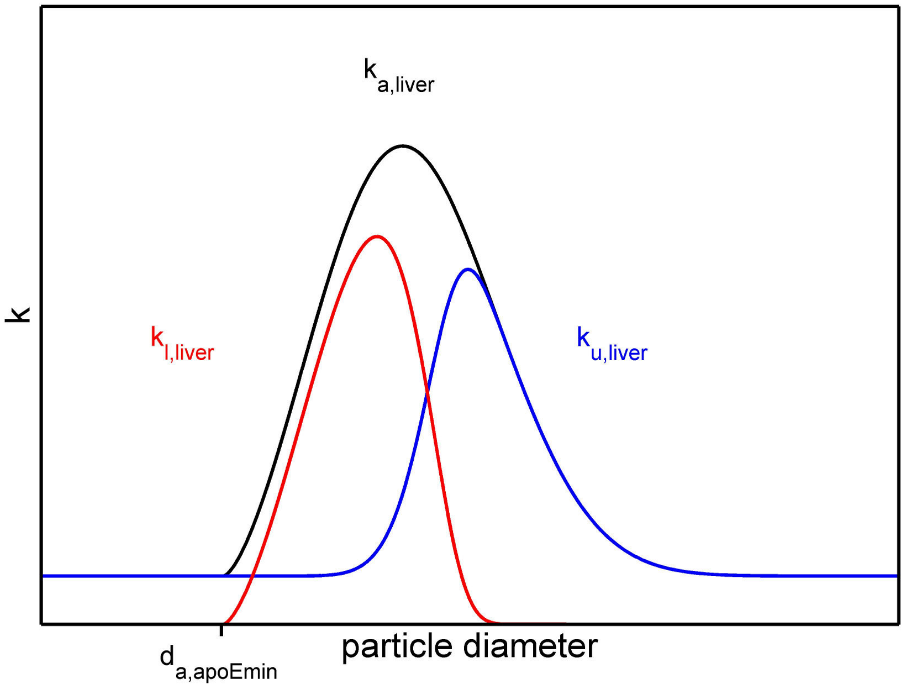

Supplement: Additional file 1 — Motivation for the equations. A complete, step-by-step motivation for the new equations introduced in this study. [file 2043-9113-1-29-S1.PDF]
